# Genetic and Flower Volatile Diversity in Natural Populations of *Origanum vulgare* subsp. *hirtum* (Link) Ietsw. in Bulgaria: Toward the Development of a Core Collection

**DOI:** 10.3389/fpls.2021.679063

**Published:** 2021-07-15

**Authors:** Marina Alekseeva, Tzvetelina Zagorcheva, Mila Rusanova, Krasimir Rusanov, Ivan Atanassov

**Affiliations:** Department of Agrobiotechnology, AgroBioInstitute, Agricultural Academy, Sofia, Bulgaria

**Keywords:** SRAP markers, SSR markers, genetic structure, GC/MS, oregano

## Abstract

We studied the genetic and flower volatile diversity in natural populations of *Origanum vulgare* subsp. *hirtum* (Link) Ietsw. in Bulgaria using simple sequence repeat (SSR) and sequence-related amplified polymorphism (SRAP) markers and gas chromatography/mass spectrometry (GC/MS) analysis of flower volatiles from individual plants. Two regions, including the Kresna Gorge and Eastern Rhodopes, typical for the species comprising eight populations and 239 individual plants were included in this study. An analysis with 11 SSR markers and eight SRAP primer combinations showed that SRAP markers were substantially more informative than the SSR markers and were further used for genetic diversity analysis. The results showed low-range to mid-range genetic differentiation between the populations with pairwise fixation index (Fst) values ranging between 0.0047 and 0.11. A total of 10 genetic clusters were identified. An analysis of the flower volatile diversity identified a total of 63 compounds with the vast majority of plants belonging to the carvacrol chemotype and just a single plant to the thymol chemotype. Large deviations were observed for individual compounds within each region as well as within the populations. Hierarchical clustering showed a clear sample grouping based on the two different regions. In addition, an in-depth analysis identified six major and 23 minor metabolite clusters. The overall data set and cluster analysis were further used for the development and testing of a simple and straightforward strategy for the selection of individual plants for the development of a core collection representing the sampled natural populations for this species in Bulgaria. The proposed strategy involves precise genetic clustering of the tested plants followed by the selection of a minimal set from each genetic cluster representing the different metabolite clusters. The selected core set was further compared with a core set extracted by the PowerCore software. A comparison of the genetic and metabolic affiliation of the members of both sets showed that the reported approach selected representatives from each genetic cluster and minor metabolic cluster, whereas some metabolic clusters were unrepresented in the PowerCore set. The feasibility and efficiency of applying the pointed strategy for the development of a core collection representing both the genetic and metabolite diversity of natural populations in aromatic and medicinal plants toward subsequent steps of selection and breeding are discussed.

## Introduction

The common name “oregano” refers to a number of species from the Lamiaceae family that produce essential oils with a characteristic scent mostly due to the high concentration of phenolic monoterpenoids like carvacrol and thymol. *Origanum vulgare* subsp. *hirtum* (Link) Ietsw. also known as Greek oregano, which has high essential oil content (Kokkini, 1997; Alekseeva et al., 2020), is among the most popular and widely used oregano spices. Despite its use for flavoring, Greek oregano is also popular in traditional medicine (Fleming, 2000) and has been a subject of numerous studies. The essential oil from *O. vulgare* subsp. *hirtum* exhibits diverse biological activities, including antibacterial (Esen et al., 2007; Stefanakis et al., 2013; Grondona et al., 2014), antifungal (Adam et al., 1998; Mancini et al., 2014; Spagnoletti et al., 2016), antioxidant (Milos et al., 2000; Sarikurkcu et al., 2015), antiparasitic (Skoufos et al., 2011), anticarcinogenic (Grondona et al., 2014), etc. The *O. vulgare* subsp. *hirtum* essential oil has been successfully used as a herbicide (Araniti et al., 2018), an antibacterial agent in the food industry (Govaris et al., 2011; Asensio et al., 2012; Al-Hijazeen et al., 2016), and a potential treatment for chronic diseases linked to oxidative stress such as Alzheimer and diabetes (Sarikurkcu et al., 2015; Vujicic et al., 2015). All these properties make the oregano essential oil an attractive product for the food, cosmetics, and pharmaceutical industries, which have been constantly looking for sources of natural and safe agents.

*Origanum vulgare* subsp. *hirtum* is distributed mainly in the Balkan Peninsula and in Turkey, and it grows at altitudes between sea level and 1,500 m, usually in dry sunny places (Ietswaart, 1980). In Bulgaria, it is distributed only in the southernmost parts of the country—in the Eastern part of the Rhodope mountains and in the Kresna Gorge in the valley of the river Struma (Konakchiev et al., 2004). Greek oregano is in the list of protected species in Bulgaria and can be collected only for personal needs from natural habitats (Stanev, 2015). However, there are risks for the natural populations due to overexploitation and a relatively limited distribution of the species in only two regions. Moreover, in recent years, the interest toward the cultivation of Greek oregano in the Eastern Rhodopes has grown considerably, which requires the breeding and production of the planting material from superior varieties. All these require measures to preserve the natural diversity of the species in Bulgaria by creating a core collection of genetic resources, which properly mirrors both the genetic and metabolite diversity in natural populations and its further application for the breeding of superior cultivars and the production of planting material.

The evaluation of the genetic diversity in natural populations requires the application of highly polymorphic low-cost markers with high discrimination power. Novak et al. (2008) developed the first set of simple sequence repeat (SSR) primers for the genus *Origanum* from expressed sequence tags (ESTs) from epidermal glands of *O. vulgare*. The set of developed SSR primers has not yet been applied in population genetic studies, and its application for intraspecific differentiation of natural populations needs to be evaluated. As an affordable alternative to the SSR markers, sequence-related amplified polymorphism (SRAP) markers, developed by Li and Quiros (2001), generate numerous fragments throughout the genome aiming for the amplification of open reading frames without the need for previous knowledge on the sequence of the studied loci. SRAP markers were successfully applied to study the genetic diversity and relationships in the genus *Origanum* in Egypt (Amar and Wahab, 2013) and Turkey (Taşcioglu et al., 2018), and were also used to evaluate the genetic diversity of other species from the Lamiaceae family (Aghaei et al., 2017; Zagorcheva et al., 2020a,b).

Although the construction of core collections representing the genetic diversity of target sets of genetic resources or natural populations has been a subject of a number of studies (Wang et al., 2007; Campoy et al., 2016; Chen et al., 2017; Miyatake et al., 2019), the construction of such core collections is largely complicated by the need to take into account the metabolic diversity together with the genetic diversity data (Liu et al., 2020). The development of core collections from natural populations is further hampered by the metabolic variation due to differences in geographical locations of natural populations and the time of sampling during the day. Thus, the development of a core collection from natural populations has to consider that the levels of metabolic variations caused by the abovementioned two factors are difficult to be evaluated and taken into account within the same first-time sampling and characterization study. Therefore, there is a clear need for the development and routine application of a simple and efficient strategy and workflow for the characterization of the genetic and metabolic diversity of natural populations toward the selection and development of core collections representing both the identified genetic and metabolic diversity of these populations.

So far, the genetic diversity in natural populations of *O. vulgare* subsp. *hirtum* in Bulgaria has not been studied with the application of modern DNA marker techniques. The breeding of new varieties has been mainly based on the grounds of morphological and morphometric traits of the selected plants from natural populations, which were subsequently tested regarding different agronomical indicators and essential oil content and composition. Konakchiev et al. (2004) studied the chemical composition of the essential oil of a plant material obtained from three populations from the Eastern Rhodopes in Bulgaria for three consecutive years. However, an in-depth evaluation of the interpopulation and intrapopulation variation, including the analysis of volatiles from individual plants, has not been carried out.

The present study focuses on the analysis of the genetic and flower volatile diversity in natural populations of *O. vulgare* subsp. *hirtum* in Bulgaria comprising the two main regions where this species grows. SSR and SRAP markers as well as gas chromatography/mass spectrometry (GC/MS) analysis of flower extracts from individual plants were applied for the characterization of the genetic and metabolic diversity of the populations. The obtained data were used for separate genetic and metabolic clustering of the studied plants and subsequent application of the cluster affiliation data for setting up a simple strategy for the selection of a core collection of individual plants well representing both the genetic and metabolic diversity of the sampled populations. The content of the selected core set was compared with that of a core set extracted by the PowerCore software. The application of such a strategy for the routine characterization and sampling of natural populations from medicinal and aromatic plants is discussed.

## Materials and Methods

### Plant Material

In the present study, the plant material was collected from eight natural populations of *O. vulgare subsp*. hirtum (Link) Ietsw. in Bulgaria—two in the Kresna Gorge region (July 2018) and six in the Eastern Rhodopes region, near the town of Ivaylovgrad (July 2019) (Table 1; Supplementary Figure 1). The plants were randomly selected covering the whole area of the population in all directions. Leaves of 239 plants were collected for the analysis of genetic diversity and the genetic structure of the populations. About 10–15 leaves from every plant were placed in 20-ml plastic containers and immediately frozen in liquid nitrogen. The samples were later transferred to the lab facilities and kept at −85°C until the extraction of genomic DNA was carried out. Also, whole inflorescences from each plant used for the collection of leaves were picked up, and four to five individual flower spikes were removed from the panicle and immersed in 4 ml ethyl acetate (Sigma) in 20-ml GC headspace vials. The GC headspace vials were tightly closed with a rubber septa and aluminum cap by using a crimp and transferred to the lab for GC/MS analysis.

**Table 1:** Global positioning system (GPS) coordinates of the eight populations of *O. vulgare* subsp. *hirtum* from the regions of the Kresna Gorge and Eastern Rhodopes used in the current study.

**Population**	**Region**	**Coordinates**
P1	Kresna Gorge	41.83309, 23.15157
P2	Kresna Gorge	41.75004, 23.11556
P3	Eastern Rhodopes	41.50879, 25.96881
P4	Eastern Rhodopes	41.50084, 26.10419
P5	Eastern Rhodopes	41.48632, 26.09905
P6	Eastern Rhodopes	41.50436, 26.1096
P7	Eastern Rhodopes	41.50488, 26.10887
P8	Eastern Rhodopes	41.5049, 26.10532

### DNA Extraction

The frozen leaf samples were ground to a fine powder in stainless steel grinding jars by using liquid nitrogen and Qiagen TissueLyser II Mill at 30 Hz for 2 min. Genomic DNA was purified according to the cetyl trimethylammonium bromide (CTAB) protocol (Murray and Thompson, 1980). Genomic DNA concentration was measured spectrophotometrically by using Nanodrop 2000 (Thermo Fisher Scientific, Waltham, MA, USA) and diluted to a final concentration of 25 ng/μl with Type I ultrapure water.

### PCR Amplification of SSR and SRAP Markers

The primer pair sequences shown in Table 2 were used for PCR amplification of SSR (Novak et al., 2008) and SRAP markers (Li and Quiros, 2001). From the forward primers used for SSR analysis, OR9, OR13, OR40, and OR44 were 5′ labeled with FAM; OR14, OR64, and OR75 were 5′ labeled with Yakima Yellow; and OR10, OR12, OR27, and OR77 were 5′ labeled with ATTO565. All reverse primers used for SRAP analysis were 5′ labeled with FAM. The combinations of primer pairs used for SRAP analysis shown in Table 2 were chosen based on the previous testing (data not shown) of 96 random primer combinations using the primers developed by Li and Quiros (2001). SRAP primers, which showed the highest number of peaks and clarity of the electropherogram, were selected for further use in the current study.

**Table 2:** Primer pairs used for sequence-related amplified polymorphism (SRAP) and simple sequence repeat (SSR) analysis.

**Marker type**	**Forward primer**		**Reverse primer**	
SRAP	ME2	TGAGTCCAAACCGGAGC	EM7	GACTGCGTACGAATTAGC
SRAP	ME3	TGAGTCCAAACCGGTAA	EM6	GACTGCGTACGAATTTTT
SRAP	ME5	TGAGTCCAAACCGGAAG	EM3	GACTGCGTACGAATTATT
SRAP	ME5	TGAGTCCAAACCGGAAG	EM6	GACTGCGTACGAATTTTT
SRAP	ME7	TGAGTCCAAACCGGTCC	EM10	GACTGCGTACGAATTTCA
SRAP	ME8	TGAGTCCAAACCGGCTG	EM8	GACTGCGTACGAATTCAC
SRAP	ME9	TGAGTCCAAACCGGTTC	EM10	GACTGCGTACGAATTTCA
SRAP	ME10	TGAGTCCAAACCGGTGA	EM1	GACTGCGTACGAATTAAA
SSR	OR9F	TTGAAGCATTGTTGGAGGTAGATG	OR9R	TCCCAACTAGGGAGAAATGTGC
SSR	OR10F	TTTGCTCCGACATCTTCAACC	OR10R	AGCCTGCTGTGTTTGGATCAG
SSR	OR12F	GCCCCTGCAGTGACTCCTAC	OR12R	AAAAAGGCTTCGGACTCGATC
SSR	OR13F	GAGAGAATCCAAGCCTCCGC	OR13R	TGAAGGAGTCCGATGTTGACG
SSR	OR14F	TGTTTGGTGGAAACCGATCC	OR14R	AGACGACGAGCTCCAATAACG
SSR	OR27F	TCAGAAACAATGAAGGCCGC	OR27R	CCGTACAGGTCAAACACCGG
SSR	OR40F	GCCCAAGGACATCCAACTTG	OR40R	CAACTGAACACCTCCCACAATG
SSR	OR44F	TCAAGGGTAGAGCTGCTGCAG	OR44R	GCTTTACGGAGGAAGAATGGG
SSR	OR64F	TCCCGCCTTCAAGAAATGAC	OR64R	AGAGAGCACGTTGATGAACCG
SSR	OR75F	CAAGAAGAATAACGGAGGAGCAG	OR75R	TGGAGAATTTCTGATGCTCGG
SSR	OR77F	TGAAGTCAGTTTGGATGATGGTG	OR77R	GTCACGTATGGAATGCACGG

PCR analysis with SSR markers was set up as multiplex reactions with primer set 1 comprising primers for loci OR09, OR10, OR14, and OR40; primer set 2 comprising primers for loci OR12, OR13, and OR75; primer set 3 comprising primers for loci OR27 and OR64; and primer set 4 comprising primers for loci OR44 and OR77. The reactions were carried out with 2-μl genomic DNA sample (total amount of 50 ng), 1 μl of each primer (forward and reverse) with a working concentration of 10 μM from each respective multiplex primer set, 10 μl MyTaq HS DNA Polymerase 2x Mix (Bioline), and Type I ultrapure water to a final volume of 20 μl. The parameters of PCR amplification included: initial denaturation for 5 min at 94°C, followed by 35 cycles at 94°C for 30 s, 58°C for 30 s, and 72°C for 30 s, with a final elongation at 72°C for 10 min.

PCR reactions for the analysis of SRAP markers were carried out as described earlier by Zagorcheva et al. (2020a). The resulting PCR products were diluted in a 1:10 ratio with Type I ultrapure water and used for fragment analysis.

### Fragment Analysis of SSR and SRAP Markers

Fragment analysis was performed on an automated capillary sequencer 3130 Genetic Analyzer (Thermo Fisher Scientific, Waltham, MA, USA) by using 36-cm long capillaries, POP-7 polymer (Thermo Fisher Scientific, Waltham, MA, USA), and GeneScan™ 500 LIZ™ as a size standard. GeneMapper Analysis Software v4.0 (Thermo Fisher Scientific, Waltham, MA, USA) was used for fragment sizing. For SSR marker analysis, alleles were reported as base pairs. The SRAP marker analysis data were collected in the range of 100–950 base pairs, and only fragments whose fluorescent signal was higher than 300 relative fluorescence units were reported and transformed into a binary matrix of 1 and 0 for each allele corresponding to their presence or absence, respectively. The generated binary matrix was manually curated for the actual presence or absence of the respective allele peaks by a visual inspection of the electropherograms.

### GC/MS Analysis of Flower Volatiles

The extraction of volatile compounds from the flower samples collected in GC headspace vials was assisted by vortexing with multi-tube vortexer (VWR) for 30 min. Then, 200-mg anhydrous sodium sulfate (Sigma) was added to each vial to remove the water contained in the samples. After vortexing for another 10 min, 1 ml of the resulting extract from each sample was transferred to a 2-ml vial for GC/MS analysis. GC/MS analyses were performed on an Agilent 7890A/5975C GC/MS system as described earlier (Zagorcheva et al., 2020a). The identification of the compounds was performed based on a comparison of their mass spectrum and the RI data with mass spectral library NIST 2008 (National Institute of Standards and Technology (NIST), Gaithersburg, MD, USA), the Robert Adams mass spectral library (Adams, 2007), and the literature data. The peak areas were calculated by following manual integration using the MSD ChemStation (Agilent Technologies, Inc., Santa Clara, CA, USA) software.

### Data Analysis

The polymorphism information content (PIC) of each SSR primer pair was calculated by using PowerMaker (Liu and Muse, 2005). Gene diversity (GD) and Shannon's information index for SRAP markers were calculated by using Popgene (Yeh, 1997). The genetic structure of the populations was analyzed with Structure 2.3.4 (Pritchard et al., 2000) where Admixture was used as an ancestry model, Length of Burnin Period was set to 100,000, and the Number of MCMC Reps after Burnin was set to 200,000. The number of presumed clusters (K) was set from 1 to 15, and 10 iterations were performed for each *K* value. Parallelization of Structure 2.3.4 calculations was achieved by using EasyParallel (Zhao et al., 2020). The most probable *K* was determined by using the method by Evanno et al. (2005) with the help of Structure Harvester (Earl and Vonholdt, 2012). The different iterations at a single *K* value were combined by using Clumpak (Kopelman et al., 2015). Analysis of molecular variance (AMOVA) and principal coordinates analysis (PCoA) were performed with GenAlEx 6.5 (Peakall and Smouse, 2006, 2012), and the genetic differentiation between the populations [pairwise fixation index (Fst)] was measured by using AFLP-SURV (Vekemans et al., 2002). Hierarchical clustering based on the SRAP marker data was performed by using Nei's genetic distance and the neighbor joining method for building dendrograms with the help of FAMD 1.31 (Schluter and Harris, 2006). The dendrogram was visualized by using Mega 6 (Tamura et al., 2013). Statistical analysis of GC/MS data, including hierarchical clustering, student's *t*-test, and ANOVA, was performed by using Mass Profiler Professional 15.1 (Agilent Technologies, Inc., Santa Clara, CA, USA). Microsoft Excel 2019 was used for building box-and-whisker plots. ArcMap 10.6.1 (Esri, Redlands, CA, USA) was used to build geographic maps of populations. Student's *t*-test (IBM SPSS statistics version 25) was used when comparing the mean of the selected parameters of a core collection with the whole data set. PowerCore 1.0 (Kim et al., 2007) was used for the development of a core collection when comparing the results from the current study with software-based methods for the development of core collections.

## Results and Discussion

### Analysis of the Genetic Diversity in Natural Populations of *O. vulgare* subsp. *hirtum* (Link) Ietsw. in Bulgaria Using SSR Markers

A total of 223 plants from 8 natural populations of *O. vulgare subsp. hirtum*, belonging to the two regions typical for the species in Bulgaria—Kresna Gorge and Eastern Rhodopes, were analyzed by means of 11 SSR primer pairs. A total of 72 alleles were scored. The most informative marker with the highest PIC value of 0.71 was OR13, and the one with the lowest PIC value was OR77 (0.25) (Supplementary Table 1). The average PIC value for all markers was 0.48. Table 3 shows the results for heterozygosity, number of alleles, and the inbreeding coefficient (Fis) for each of the eight populations. The mean expected heterozygosity (He) and the mean observed heterozygosity (Ho) were 0.482 and 0.448, respectively, showing a moderate level of genetic diversity. The mean number of alleles (Na) and the mean number of effective alleles (No) were 3.818 and 2.147, respectively. All values of Fis were close to 0, thus implying random mating. The negative value for population P1 indicated an excess of heterozygosity, which was anticipated since the observed heterozygosity for P1 was higher than the expected. The pairwise Fst between the populations ranged from 0.018 to 0.09 with a mean value of 0.047 suggesting a low degree of genetic differentiation between the populations.

**Table 3:** Mean heterozygosity, number of alleles, and inbreeding coefficient (Fis) for each of the eight studied populations based on the data from an analysis of 223 *Origanum vulgare* subsp. *hirtum* plants with 11 SSR primer pairs.

**Population**	**He (Expected heterozygosity)**	**Ho (Observed heterozygosity)**	**Na (Number of alleles)**	**Ne (Number of effective alleles)**	**Fis (Inbreeding coefficient)**
P1	0.453	0.468	3.818	2.234	−0.024
P2	0.504	0.461	4.091	2.315	0.066
P3	0.542	0.516	5.000	2.357	0.038
P4	0.462	0.382	3.273	1.975	0.129
P5	0.480	0.456	3.818	2.096	0.047
P6	0.450	0.411	3.000	1.954	0.090
P7	0.525	0.487	4.000	2.244	0.082
P8	0.441	0.405	3.545	2.003	0.074
Mean	0.482	0.448	3.818	2.147	0.063

The performed PCoA analysis (Supplementary Figure 2) based on the analysis with 11 SSR primer pairs showed a lack of clear clustering of the samples belonging to the two separate regions — the samples from the Kresna Gorge region assorted with the samples from the Eastern Rhodopes region, which corresponded well to the low pairwise Fst values. In our study, 223 individuals from eight natural populations of *O. vulgare subsp. hirtum* were analyzed at 11 loci generating a total of 72 alleles. The poor differentiation between the populations may be the result of an insufficient number of alleles. Additionally, the SSR primer pairs used in this study are derived from ESTs, which may result in the detection of a lower number of polymorphisms, compared to genomic SSRs in non-coding regions. The detection of lower polymorphism using EST-SSRs in comparison to nuclear SSRs has been reported in studies including diverse plant species like sugarcane (Parthiban et al., 2018), wheat (Yang et al., 2005), cucumber (Hu et al., 2011), etc.

The application of SSR markers for analysis of within species genetic diversity requires selection of loci with high genetic diversity in populations and a high number of alleles per locus. The set of EST-derived SSR markers used in the present study are currently the only available SSR markers in the genus Origanum (Novak et al., 2008). Further efforts are needed for the identification of highly polymorphic loci that are suitable for within-species analysis of genetic diversity in *O. vulgare* natural populations.

### Analysis of the Genetic Diversity in Natural Populations of *O. vulgare* subsp. *hirtum* in Bulgaria Using SRAP Markers

**Figure 1:**
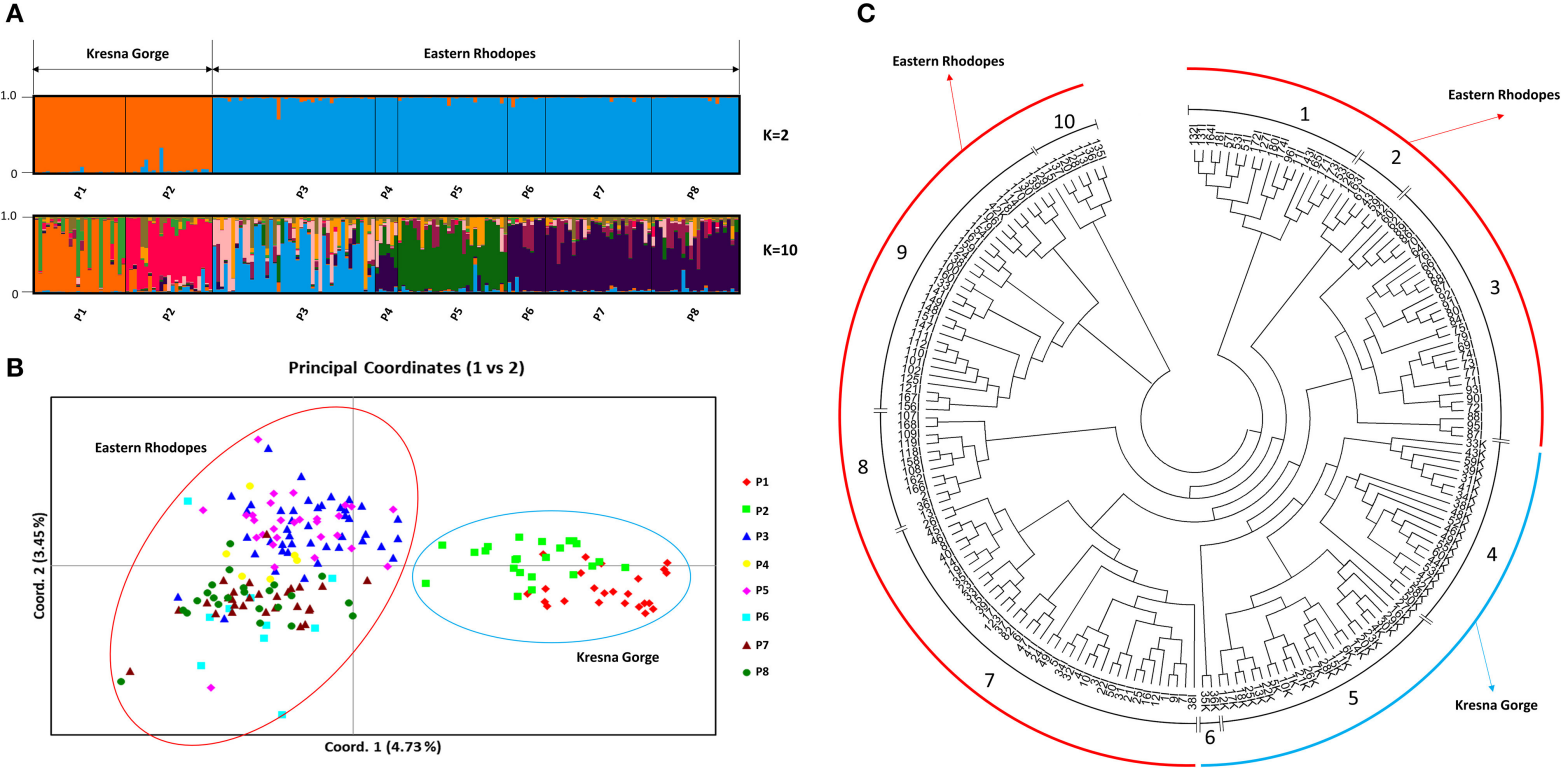
Genetic structure of eight natural *Origanum vulgare* subsp. *hirtum* (Link) Ietsw. populations in Bulgaria based on sequence-related amplified polymorphism (SRAP) marker data: **(A)** A bar plot representing the genetic structure at *K* = 2 and *K* = 10, populations P1 and P2 belong to the Kresna Gorge region, and P3–P8 belong to the Eastern Rhodopes region. **(B)** Principal coordinates analysis (PCoA), populations P1 and P2 belong to the Kresna Gorge region, and P3–P8 belong to the Eastern Rhodopes region. **(C)** Neighbor-joining dendrogram. Samples with names ending with the letter *K* belong to Kresna Gorge. Samples with names ending with the letter I belong to Eastern Rhodopes.

In the present study, 186 plants from eight natural populations of O. vulgare subsp. hirtum, belonging to the two regions typical for the species in Bulgaria—Kresna Gorge and Eastern Rhodopes, were analyzed by using eight SRAP primer pairs. The performed SRAP analysis identified a total of 470 polymorphic alleles. Unlike the SSR marker analysis, the use of the SRAP marker data for PCoA clustering showed a clear separation of the samples from the two regions as well as clustering of individual populations within the regions (Figure 1B). An analysis of the genetic structure of all 186 samples using the Structure software showed that the most probable value of *K* according to the Evanno method (Evanno et al., 2005) was *K* = 2 (Supplementary Figure 3). Figure 1A shows a bar plot representing the genetic structure at *K* = 2 for the analyzed eight populations comprising the two analyzed regions. According to Figure 1A, the observed genetic structure at *K* = 2 is based on the diversity between the two regions. However, as indicated by the PCoA analysis (Figure 1B), a genetic substructure exists in each of the two regions. The performed hierarchical clustering (Figure 1C) based on Nei's genetic distances allowed the identification of 10 genetic clusters. Visualization of the genetic structure of the populations at *K* = 10 (Figure 1A) showed that within each population a noticeable heterogeneity of the genetic structure is observed among the individual samples, which proves that random selection of plants from the individual populations for the development of genetic resource collections may result in underrepresentation of the actual genetic diversity.

Table 4 shows the results from pairwise Fst comparison among all populations as a measure of their genetic differentiation. The results for Fst showed low-range to mid-range differentiation between the populations. In general, the values between any of the populations from the Eastern Rhodopes region were lower than those between populations P1 and P2 in the Kresna Gorge region. That is to say, the differentiation between the populations from the Eastern Rhodopes region is lower than the differentiation between the populations from the Kresna Gorge region. As expected, the highest Fst values were scored between the populations belonging to different regions with the highest being 0.1101 between P1 and P5. The lowest Fst of 0.0047 was scored between P7 and P8 belonging to the Eastern Rhodopes region. According to the data, the lowest differentiation was observed between populations P6, P7, and P8, which are also closely located on the PCoA graph (Figure 1B). The results from AMOVA showed that a significant part of the observed variation was due to the variation among individuals within the populations (85%) while 8% was due to the variation among populations and 6% between regions (Table 5).

**Table 4:** Pairwise comparison of the Fst values based on the data from an analysis of 186 *O. vulgare* subsp. *hirtum* plants from eight natural populations using eight SRAP primer pairs.

	**P1**	**P2**	**P3**	**P4**	**P5**	**P6**	**P7**	**P8**
P1	0.0000							
P2	0.0780	0.0000						
P3	0.0954	0.0690	0.0000					
P4	0.0988	0.0609	0.0268	0.0000				
P5	0.1101	0.0799	0.0504	0.0244	0.0000			
P6	0.1064	0.0917	0.0669	0.0448	0.0754	0.0000		
P7	0.0989	0.0830	0.0496	0.0252	0.0592	0.0197	0.0000	
P8	0.1008	0.0837	0.0461	0.0218	0.0561	0.0219	0.0047	0.0000

*Populations P1–P2 belong to the Kresna Gorge region. Populations P3–P8 belong to the Eastern Rhodopes region*.

**Table 5:** Summarized data from the analysis of molecular variance (AMOVA) of 186 *O. vulgare* subsp. *hirtum* plants from eight natural populations using 8 SRAP primer pairs.

**Source**	**Df**	**SS**	**MS**	**Est. Var**.	**%**
Among regions	1	468.125	468.125	4.033	6%
Among populations	6	1015.881	169.313	5.201	8%
Within populations	178	9661.043	54.276	54.276	85%
Total	185	11145.048		63.510	100%

Katsiotis et al. (2009) studied the genetic diversity of *O. vulgare* subsp. *hirtum* in Greece using random amplified polymorphic DNA (RAPD) markers. The results revealed that genetic variability was distributed mainly within populations, however, there was also noteworthy genetic differentiation between different geographical localities. The results from the present study show that, in general, the genetic diversity of *O. vulgare* subsp. *hirtum* in Bulgaria is largely determined by the two separate regions characteristic of this species as the main part of the variation within each region is mainly due to a variation within the populations.

### Flower Volatile Diversity

Gas chromatography/mass spectrometry analysis of flower extracts from 239 single *O. vulgare* subsp. *hirtum* plants in eight natural populations resulted in the identification of a total of 63 compounds, representing an average of 84% of the total area of the chromatograms. Despite the relatively large number of identified compounds, only 10, including α-thujene, α-pinene, myrcene, α-terpinene, p-cymene, γ-terpinene, thymoquinone, caryophyllene (E-), β-bisabolene, and carvacrol, of them had a mean value of more than 1% (Supplementary Tables 2–5; Supplementary Figure 4). The results confirmed that the populations of *O. vulgare* subsp. *hirtum* in Bulgaria are mainly of carvacrol chemotype, which was the predominant compound in 238 of 239 tested samples with an average value of 56.4% ± 7.3%. The mean value of thymol, which is the major compound in *O. vulgare* subsp. *hirtum* with thymol chemotype, was only 0.45% ± 2.8%. In a single sample of population P3 from the Eastern Rhodopes region, a change in the chemotype from carvacrol to thymol was observed, with a content of thymol of 43.66% and only 4.14% of carvacrol. The observed change in the chemotype is most likely due to gene mutations related to terpenoid synthesis or changes in their expression as a result of genetic or epigenetic factors.

ANOVA showed that a total of 45 compounds vary statistically significantly between the regions, of which 38 at *p* < 0.01 (Supplementary Table 2). Carvacrol showed statistically significant higher content in the samples from the Kresna Gorge region with an average value of 59.3 and 55.72% for the Eastern Rhodopes region. Apart from carvacrol, the only compounds for which higher values were scored in the Kresna Gorge region and with a percentage more than 1%, were thymoquinone and β-bisabolene, comprising 3.04 and 1.2%, respectively for the Kresna Gorge region, and 0.99 and 0.78% for the Eastern Rhodopes region. A total of seven compounds, such as α-thujene, α-pinene, myrcene, α-terpinene, p-cymene, γ-terpinene, and caryophyllene (E-), with a percentage of more than 1% were reported with higher amounts in the samples from the Eastern Rhodopes region, with amounts of 2.7, 1.05, 1.95, 1.06, 5.24, 9.33, and 2.43% for the Eastern Rhodopes region, and 0.98, 0.42, 0.73, 0.25, 4.39, 4.37, and 1.4% for the Kresna Gorge region, respectively. The differences in the amounts of certain compounds between the two regions may be due to genetic factors as well as regional and climatic factors, including the fact that the samples from the two regions were analyzed in two consecutive years.

Close inspection of the results shown in Supplementary Table 2 reveals significant variation in the compounds between individual plants within each region. For example, γ-terpinene varies from 0.33 to 14.8% in particular samples in the populations from the Kresna Gorge region and from 0.08 to 24.74% in the populations from the Eastern Rhodopes region. The same applies to caryophyllene (E-) and thymoquinone. Caryophyllene (E-) ranges between 0.15 and 3.01% in individual samples in the populations from the Kresna Gorge region and between 0.49 and 9.46% in the populations from the Eastern Rhodopes region and for thymoquinone the values for the populations in the Kresna Gorge region are in the range between 0.61 and 13.22% and for the Eastern Rhodopes region—from 0 to 6.49%. Similarly, large variations in the abundance of compounds were also observed within the populations in each region (Supplementary Table 3). Population's diversity of the species depends on environmental factors and life history characteristics (Sheidai et al., 2014; Matsumoto et al., 2020). A study on plant species containing high valuable compounds is of great importance, increasing the status of herbal industry (Goodman, 2020; Miao et al., 2020). Therefore, simply random selection of plants from the individual populations in the two regions for the development of a collection of genetic resources reflecting the metabolite diversity in populations might lead to a misrepresentation of the actual volatile diversity.

**Figure 2:**
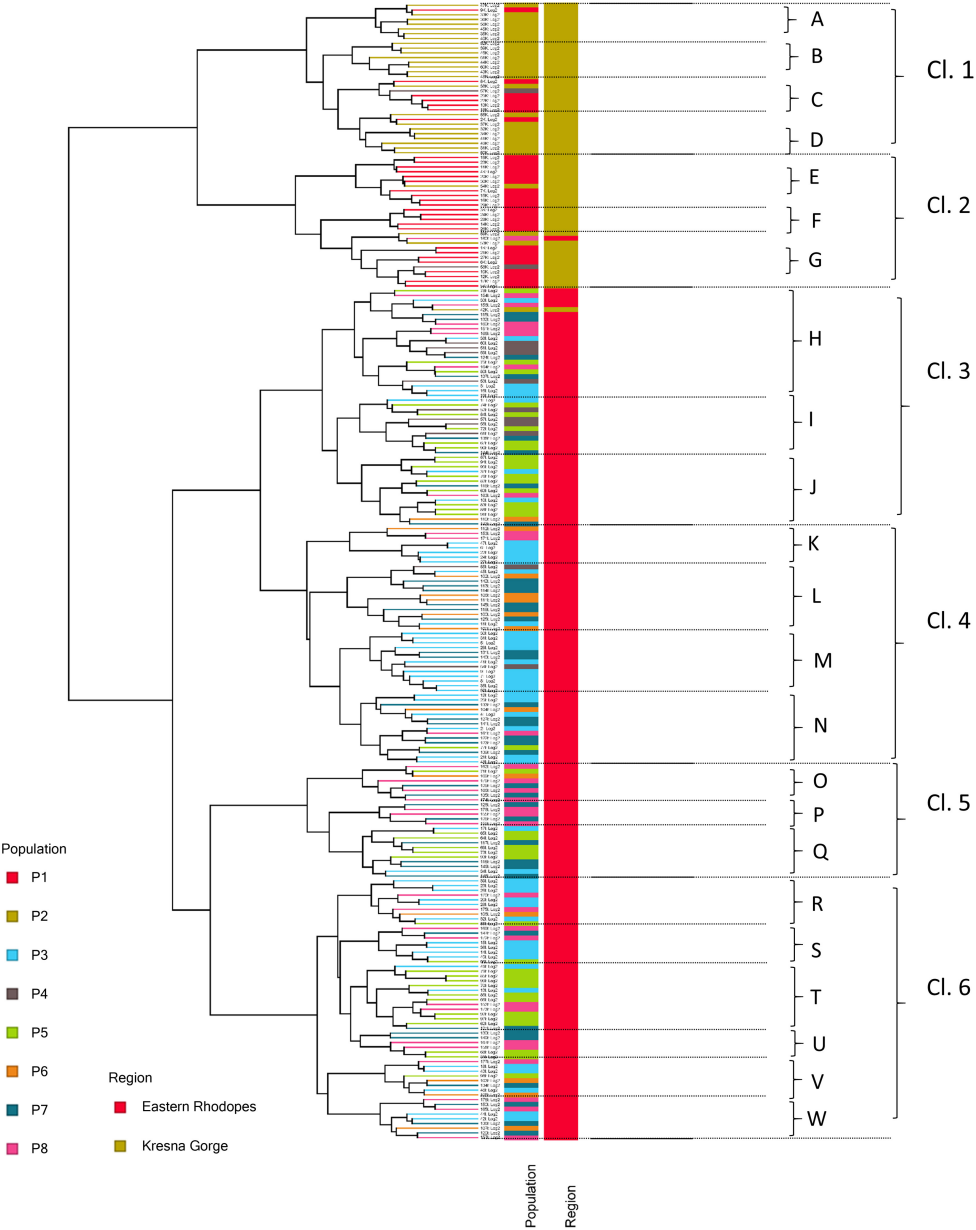
Hierarchical clustering of 239 samples of *O. vulgare* subsp. *hirtum* (Link) Ietsw., based on the relative amounts of 63 compounds identified in flower extracts of single plants in eight populations and two regions in Bulgaria. Cl.1–Cl.6 and A–W designate major and minor metabolite clusters, respectively. Populations P1 and P2 belong to the Kresna Gorge region, and P3–P8 belong to the Eastern Rhodopes region.

The GC/MS analysis data obtained in this study were further used for hierarchical clustering (Figure 2). As can be seen from Figure 2, two main clusters of samples were formed corresponding to the two individual studied regions—the Kresna Gorge and the Eastern Rhodopes. Largely, the samples from the cluster corresponding to the Kresna Gorge region gathered reflecting their segregation by populations while for the samples from the Eastern Rhodopes region clustering based on the population origin was not observed. Further, 6 metabolic sub-clusters designated as Cl.1–Cl.6 were identified, and within each subcluster a total of 23 minor metabolic clusters designated with letters A–W were also identified. The data for the mean value and the range of variation of the identified compounds for each cluster are presented in Supplementary Tables 4, 5. The obtained grouping of the samples into separate clusters with a relatively similar metabolic profile provides a possibility to select individual representatives from each cluster in order to create a collection that reflects the metabolic diversity in the populations.

Until now, there are a few studies based on the geographic variation and diversity of *O. vulgare* subsp. *hirtum*, and most of them are concentrated on the differences in the essential oil content and in the morphology-in Greece (Vokou et al., 1993; Gavalas et al., 2011), Italy (Tuttolomondo et al., 2012), and Albania (Elezi et al., 2013). The present study adds more details to what the actual volatile diversity in natural populations of this species is by analyzing flower extracts from individual plants in eight populations comprising two regions in Bulgaria.

### Construction of a Core Collection

**Figure 3:**
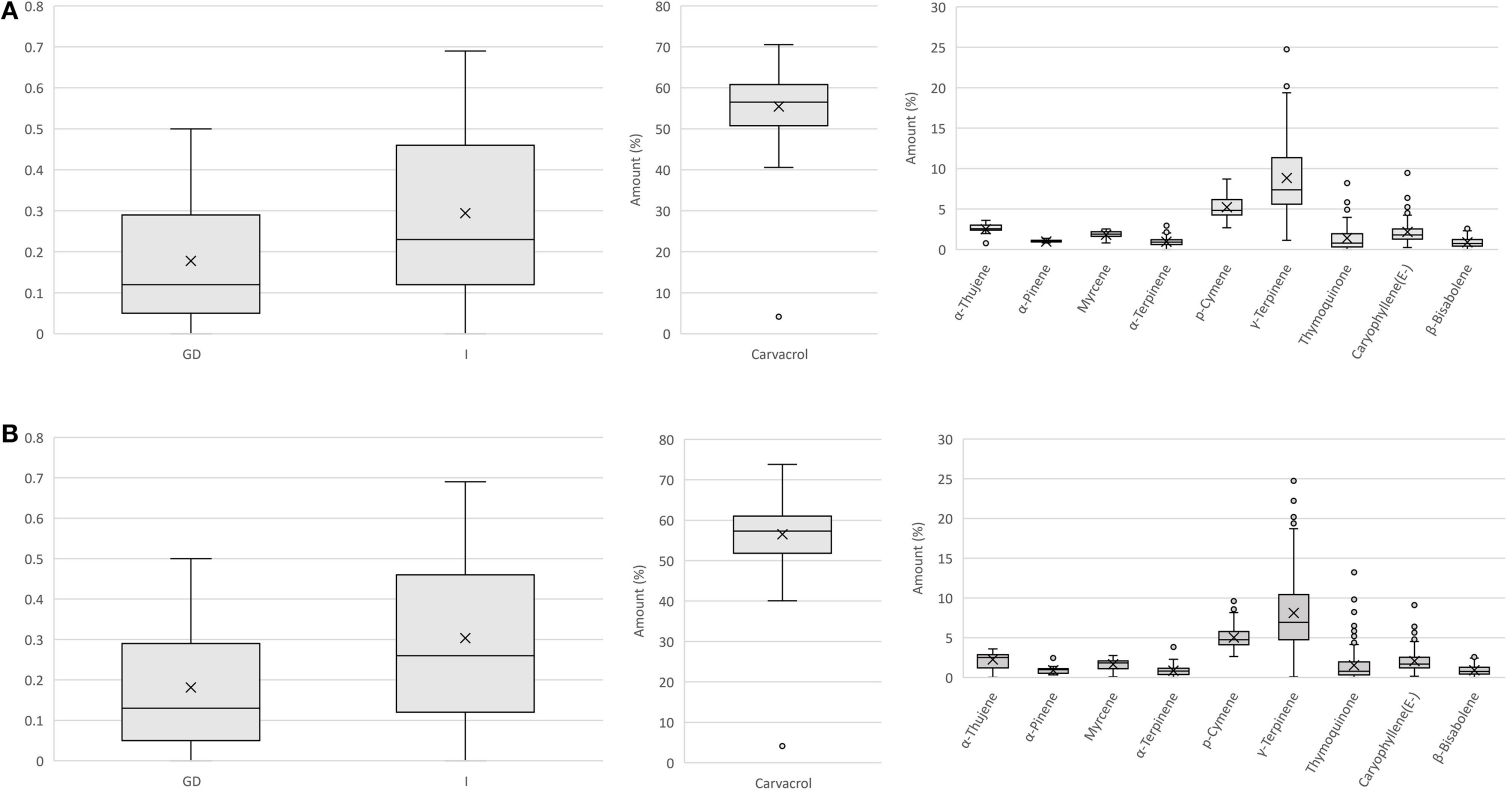
Box-and-whiskers plot showing the distribution of data for Nei's Gene diversity (GD), Shannon's information index (I), and amounts of the 10 compounds with a percentage of more than 1% in flower extracts for: **(A)** the selected core set of 78 plants and **(B)** the whole set of 186 plants analyzed by both SRAP markers and gas chromatography/mass spectrometry (GC/MS) analysis of flower extracts.

We used the results from the analysis of the genetic and flower volatile diversity in natural populations of *O. vulgare* spp. *hirtum* in Bulgaria for the development and testing of a simple strategy and workflow for the construction of a core collection for this species representing both the genetic and metabolic diversity of the sampled natural populations. One of the main challenges for the construction of such core collections is related to the inability of such studies to determine and take into account the impact of different environmental conditions related to the development and sampling of the tested plants from different populations located in remote areas as well as the difficulties in joint processing of the genetic and metabolic data. This has to be especially taken into consideration for the construction of core collections from natural populations of medicinal and aromatic plants with complex metabolite composition and often very large variations of the levels in single metabolites of interest. Therefore, simply combining the obtained genetic and metabolic data and their processing using the currently available core collection software products could produce results with mis-collection of valuable accessions. That is why we used a strategy based on separate genetic and metabolic clustering of all tested plants from the different populations. In the next step, the affiliation of the plants from each genetic cluster to the identified metabolic clusters was considered. Finally, the core collection was constructed through the selection and combining of a minimal set of plants representing the different metabolic clusters, which are present in each genetic cluster (Supplementary Figure 5). The results from the pointed steps of the construction of a core collection are illustrated in Supplementary Table 6. The data from the genetic clustering shown in Figure 1C were transformed into a table where each sample starting from genetic cluster 1 was transferred to a row in the table with a separate row designating the affiliation of the sample to a particular metabolic cluster. The first sample from each genetic cluster belonging to a different metabolic cluster was selected for the construction of a core set for the genetic cluster. After that, the selected core sets were combined to construct the entire core collection. As a result, a core collection of 78 plants of 186 totally tested plants was selected. The selected core collection represents 41.9% of the whole set of initially characterized plants. A comparison of the distribution of data for Nei's gene diversity, Shannon's information index, as well as for the amounts of the 10 compounds with a percentage of more than 1% in the flower extracts showed a remarkably similar pattern for both the core set (Figure 3A) and all studied plants (Figure 3B), with no significant difference between the average values following *t*-test analysis. Similar approach has recently been proposed for the medicinal plant *Angelica biserrata* (RH Shan and CQ Yuan) CQ Yuan and RH Shan (Liu et al., 2020). We further compared our results with the results obtained with PowerCore, which is a dedicated software for the development of core collections. Using the heuristic method of PowerCore, we extracted a core collection of 54 plants representing 29% of all analyzed samples. The *t*-test analysis showed no statistically significant differences between the means for Nei's gene diversity, Shannon's information index, and the abundances of compounds with a percentage of more than 1% with exception of the major compound Carvacrol where means were significantly different with 54 ± 10% Carvacrol in the core set vs. 57 ± 8% in the whole data set. The selected set of 54 plants selected by PowerCore are shown in Supplementary Table 6, in parallel with the set of 78 plants selected using the method presented in the current study. A comparison of the genetic and metabolic affiliation of the plants from both sets demonstrated that PowerCore has not selected representatives of several minor metabolic clusters allocated within the different genetic clusters, and has not selected representatives of the less abundant genetic cluster 6, Supplementary Table 6. Although differences in the metabolome observed in natural populations could be due to environmental factors related to the location of the target populations or period of sampling, such differences could also be due to genetic factors, which cannot be detected by using the applied genetic markers. Therefore, the selection of an initial broader set of plants representing the overall metabolic diversity of plants affiliated to one and the same genetic cluster is of high importance for inclusion in the selected core set of potentially valuable accessions, which although genetically closer have distinct metabolite composition and differ in the content of one or few valuable compounds. Therefore, in spite of resulting in a higher portion of selected plants, the applied approach will be particularly suitable and useful for the selection of core sets from the members of natural populations of medicinal and aromatic plants for which the value of selected accessions is largely determined by the levels of accumulation of a small set of metabolites.

Based on the obtained results, we propose the described here strategy for the development of a core collection for *O. vulgare* subsp. *hirtum* to be applied as a routine for other medicinal and aromatic plants, including protected plant species. The related workflow should include several well-targeted steps carried out during the same vegetation season. The first step should be carried out at the beginning of vegetation and includes the collection of leaf samples from natural populations of randomly selected individual plants as well as the labeling of the plants with wrap-around tags. Our experience shows that the wrap-around tags remain in place within the same vegetation season, and the respective plants can be recognized during a later visit facilitated by the respective global positioning system (GPS) data. Following SRAP analysis and identification of groups of plants with a similar genetic constitution, a second visit to the natural populations should be carried out during the period of active accumulation of metabolites of interest, which, for most of the plants, overlap the active flowering period. Then, the samples from organs and tissues accumulating the metabolites of interest should be collected and subjected to metabolite analysis. This second visit could also be used for an additional increase in the number of samples from particular populations showing higher genetic diversity and heterogeneity of the population structure. By following the above pointed data processing, the plants to be included in the core collection could be identified within the same vegetation period. Depending on the plant species and planned method for the establishment of a core collection, seeds or plant samples for *in vitro* or *in vivo* multiplication should be collected for subsequent growing in experimental fields and a more detailed characterization of the core collection. Considering the fact that SRAP markers could be universally applied to a wide range of plant species, and the methods for general metabolite analysis [GC/MS and/or liquid chromatography/MS (LC/MS)] are largely available for most of the medicinal and aromatic plants, we propose that the pointed strategy could also be routinely applied for the development of core collections from natural populations of a wide range of medicinal and aromatic plants, which are of interest for breeding and (semi-) industrial cultivation.

## Conclusion

The results from the present study show that SRAP markers proved to be a reliable and an affordable tool for the analysis of the genetic diversity and population structure in natural populations of *O. vulgare* spp. *hirtum* (Link) Ietsw. The currently available EST-derived SSR markers do not provide enough resolution to allow for genetic differentiation of the populations, and further efforts will be necessary to identify SSR markers, which are suitable for population genetics studies in this species. The results from SRAP analysis showed low-range to mid-range genetic differentiation between the studied *O. vulgare* subsp. *hirtum* populations in Bulgaria and pointed out that the genetic diversity is determined by the two separate regions characteristic of the species as the main part of the variation within each region is mainly due to a variation within the populations. GC/MS analysis of flower volatiles from individual plants confirmed that the populations in Bulgaria belong to the carvacrol chemotype although single plants belonging to the thymol chemotype could occasionally be discovered. Large variations of the flower volatiles were observed between the two regions as well as among and within the populations. Based on the genetic diversity data generated with SRAP markers and the metabolic diversity evaluation *via* GC/MS analysis of flower volatiles, a simple and straightforward strategy for the development of a core collection representing both the genetic and metabolic diversity of the sampled plants was established, which prevents potential failure to identify and select plants with a unique metabolite composition. The strategy employs separate genetic and metabolic clustering of the analyzed plants and the combination of the results for the selection of plants representing all identified genetic and metabolite clusters. The efficiency of the proposed strategy for the selection of a core set was further demonstrated by a comparison with the application of the dedicated software PowerCore, which showed that the PowerCore elaborated set left unrepresented a number of minor metabolic clusters within the identified genetic clusters. Accordingly, we propose that the same strategy could be routinely applied for the development of core collections mirroring the genetic and metabolite diversity in natural populations of other medicinal and aromatic plant species, where the value of selected plants is often related to the abundancies of a small set of metabolites.

## Data Availability Statement

The original contributions presented in the study are included in the article/Supplementary Material, further inquiries can be directed to the corresponding author/s.

## Author Contributions

MA was the main person involved in the study, performing all experimental work which later became the main part of her Ph.D. Thesis. TZ contributed to the analysis of SRAP marker data. MR was involved in SSR and SRAP fragment analysis by capillary electrophoresis and analysis of SSR data. KR was the supervisor of MA during her experimental work and contributed to all data analysis. IA was involved in the whole experiment and workflow design. All authors contributed to the article and approved the submitted version.

## Conflict of Interest

The authors declare that the research was conducted in the absence of any commercial or financial relationships that could be construed as a potential conflict of interest.

## Supplementary Material

Supplementary Figure 1 Geographic maps with marked locations of the studied populations.

Supplementary Figure 2 Principal coordinates analysis (PCoA) based on data from the analysis of 223 *Origanum vulgare* subsp. *hirtum* (Link) Ietsw. plants from eight natural populations with 11 simple sequence repeat (SSR) primer pairs.

Supplementary Figure 3 Detection of the number of genetic clusters *K* by using the DeltaK method in Evanno et al. (2005).

Supplementary Figure 4 Gas chromatography/mass spectrometry (GC/MS) chromatograms of flower volatiles from: **(A)** a plant representing the carvacrol chemotype and **(B)** the only plant in the study representing the thymol chemotype. Numbers above peaks indicate the compounds with amount more than 1% in any of the particular samples: (1) α-thujene, (2) α-pinene, (3) sabinene, (4) myrcene, (5) α-terpinene, (6) p-cymene, (7) γ-terpinene, (8) cis-sabinene hydrate, (9) carvacrol methyl ether, (10) thymoquinone, (11) thymol, (12) carvacrol, (13) caryophyllene(E-), and (14) β-bisabolene.

Supplementary Figure 5 Workflow applied for the development of a core collection in the current study based on in parallel evaluation of the genetic and metabolic diversity in natural populations of *O. vulgare* subsp. *hirtum* (Link) Ietsw. in Bulgaria.

Supplementary Table 1 Polymorphism information content (PIC) values for the 11 simple sequence repeat (SSR) markers.

Supplementary Table 2 Percentage content and range of variation of 63 compounds in extracts of *Origanum vulgare* subsp. *hirtum* (Link) Ietsw. inflorescences for the two studied regions (KG, Kresna Gorge; ER, Eastern Rhodopes).

Supplementary Table 3 Percentage content and range of variation of 63 compounds in extracts of *O. vulgare* subsp. *hirtum* (Link) Ietsw. inflorescences for the eight studied populations.

Supplementary Table 4 Percentage content and range of variation of 63 compounds in extracts of *O. vulgare* subsp. *hirtum* (Link) Ietsw. inflorescences for the six major metabolic clusters marked as C1–C6 in Figure 2.

Supplementary Table 5 Percentage content and range of variation of 63 compounds in extracts of *O. vulgare* subsp. *hirtum* (Link) Ietsw. inflorescences for the 23 minor metabolic sub-clusters marked as A–W in Figure 2.

Supplementary Table 6 Selection of plants for the development of a core collection based on their affiliation to genetic and metabolic clusters. First row shows the name of the sampled plants. Second and third rows show the affiliation of the respective plant to a particular genetic (2nd row) and metabolic (3rd row) cluster, numbered according to Figures 1C, 2. P1–P8 indicate the population origin of the respective sample. Symbol “X” indicates the plants included in the core collection using the method presented in the current study. Symbol “Y” indicates the plants selected by PowerCore. Red-filled cells in the row labeled as “Missed by PowerCore” indicate samples, which belong to the metabolic clusters that were not represented within the respective genetic cluster in the core set extracted by PowerCore. Blue-filled cells in the eighth row show names of the genetic and metabolic clusters unrepresented in the core set extracted by PowerCore.
