# Supplementary figures and images for: Genetic and Flower Volatile Diversity in Natural Populations of Origanum vulgare subsp. hirtum (Link) Ietsw. in Bulgaria: Toward the Development of a Core Collection
Source: Front Plant Sci. 2021 Jul 15;12:679063. doi: 10.3389/fpls.2021.679063 (PMC8320660; doi:10.3389/fpls.2021.679063)

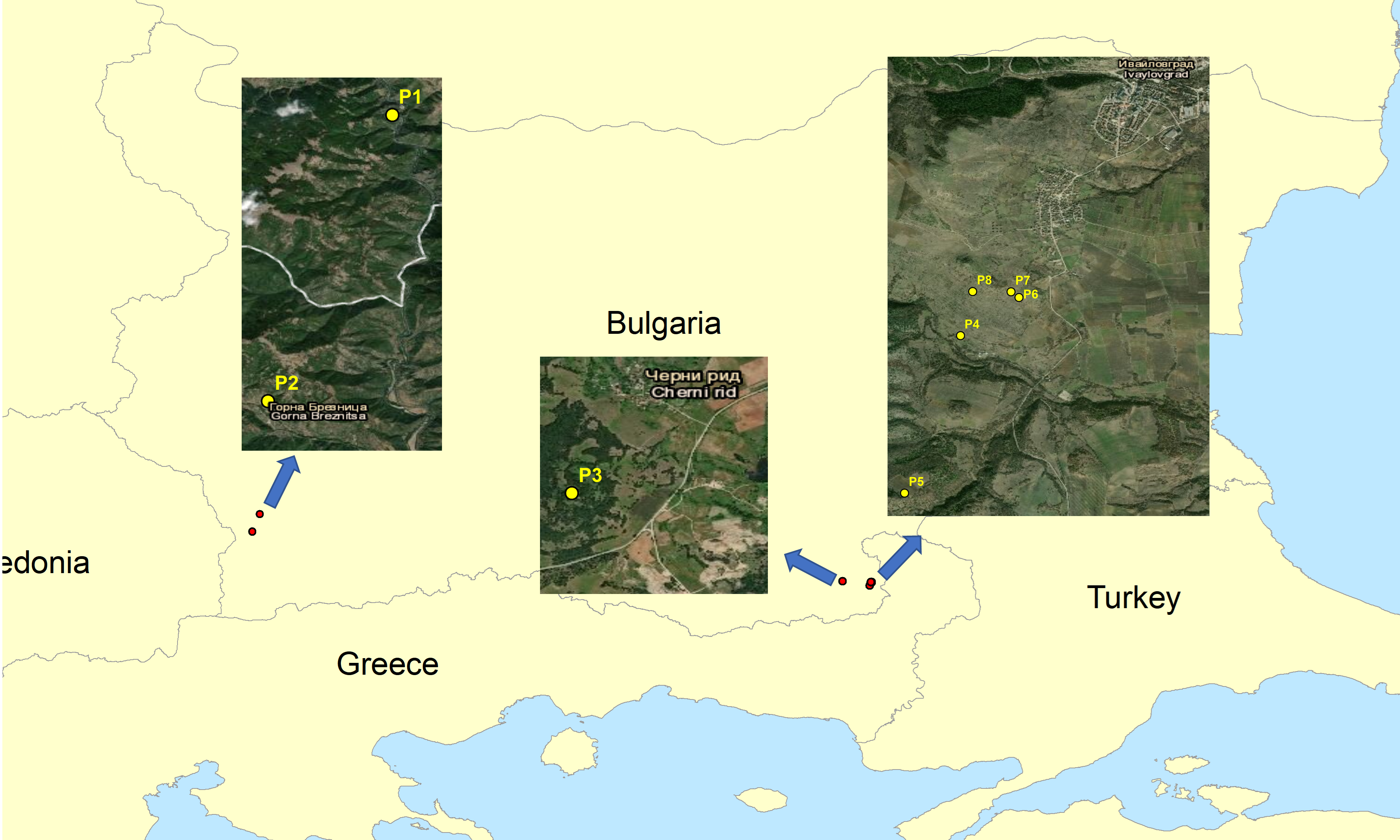

Supplement: Supplementary Figure 1 — Geographic maps with marked locations of the studied populations. [file Figure_1.TIF]

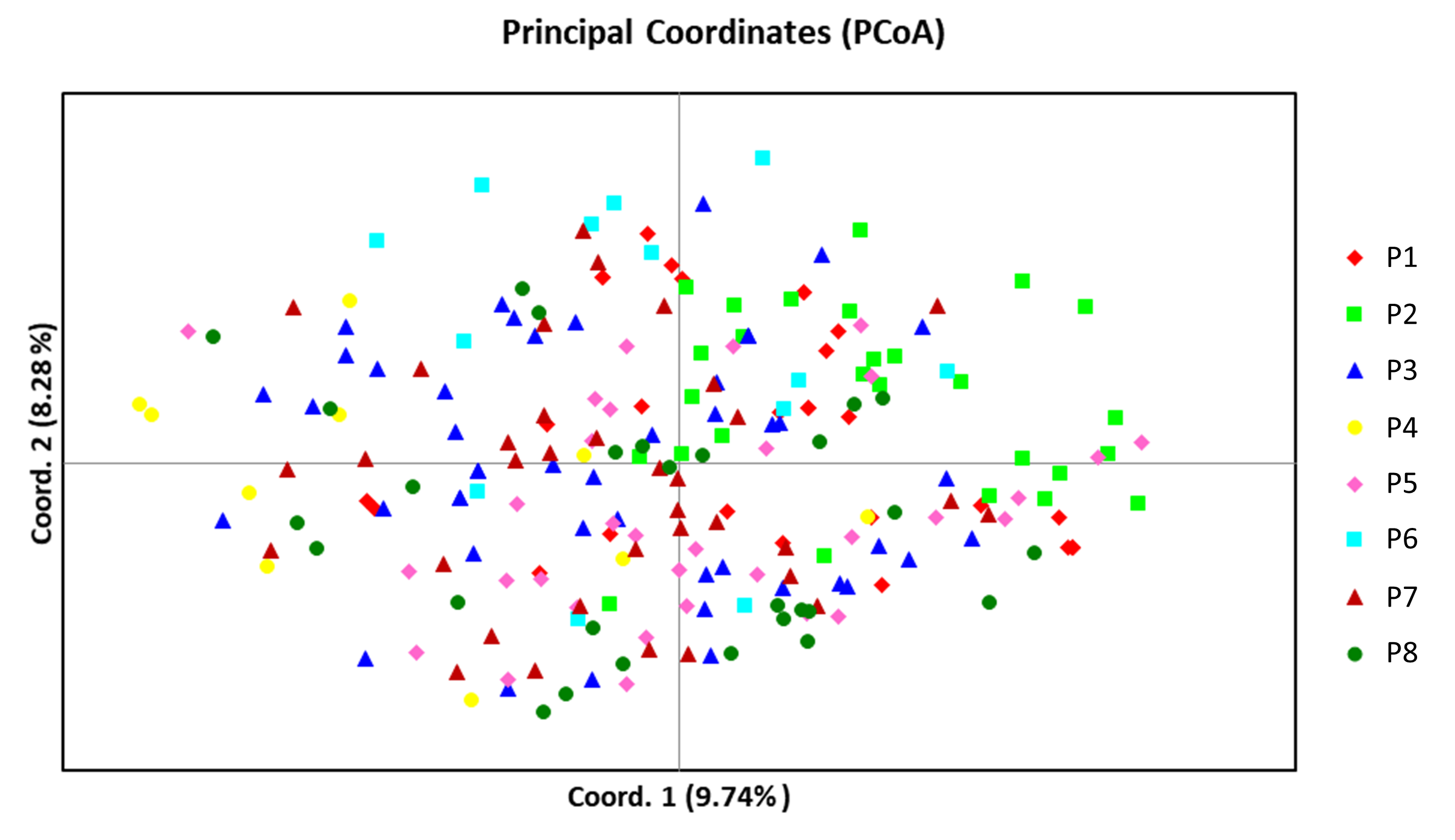

Supplement: Supplementary Figure 2 — Principal coordinates analysis (PCoA) based on data from the analysis of 223 Origanum vulgare subsp. hirtum (Link) Ietsw. plants from eight natural populations with 11 simple sequence repeat (SSR) primer pairs. [file Figure_2.TIF]

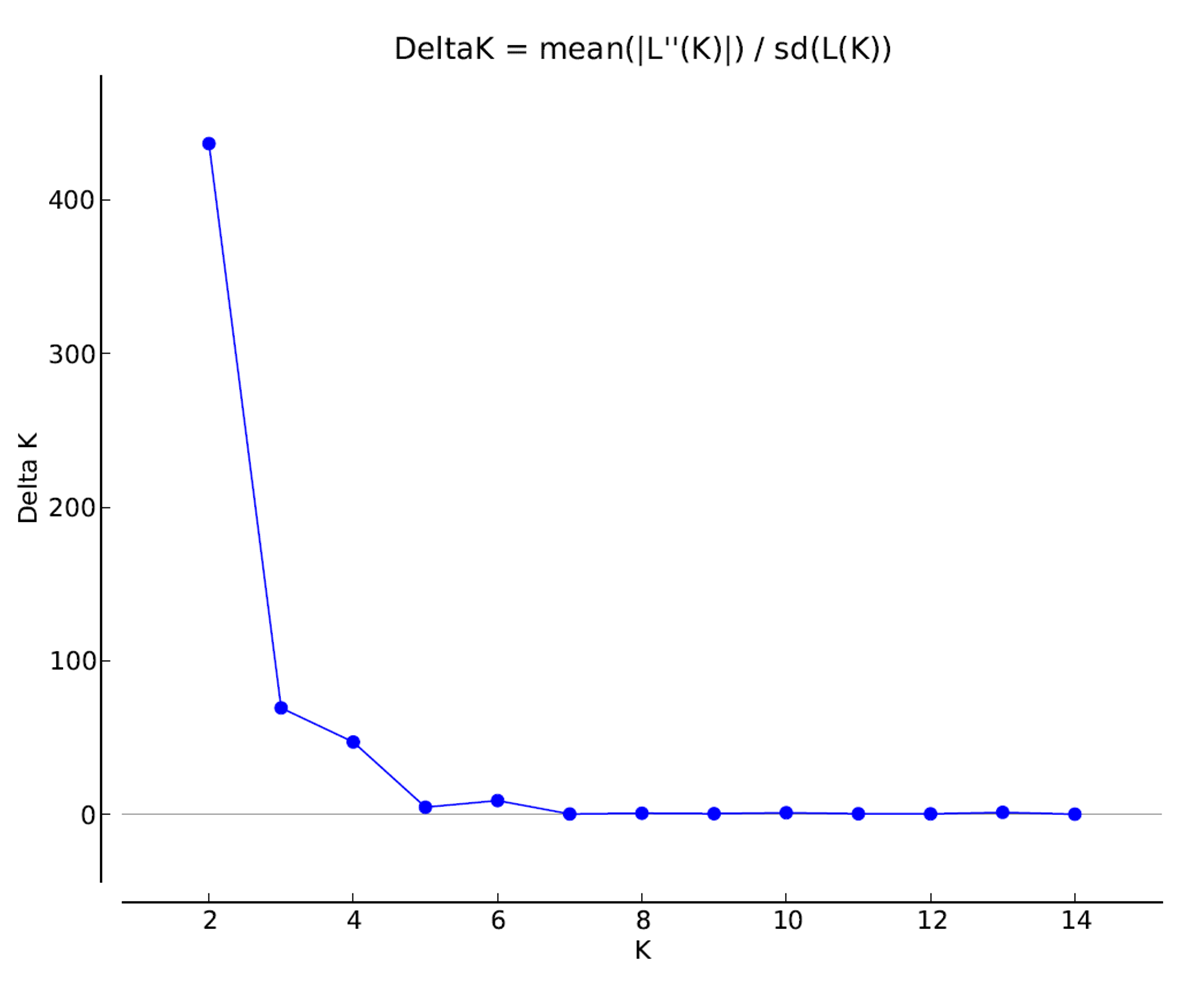

Supplement: Supplementary Figure 3 — Detection of the number of genetic clusters K by using the DeltaK method in Evanno et al. (2005). [file Figure_3.TIF]

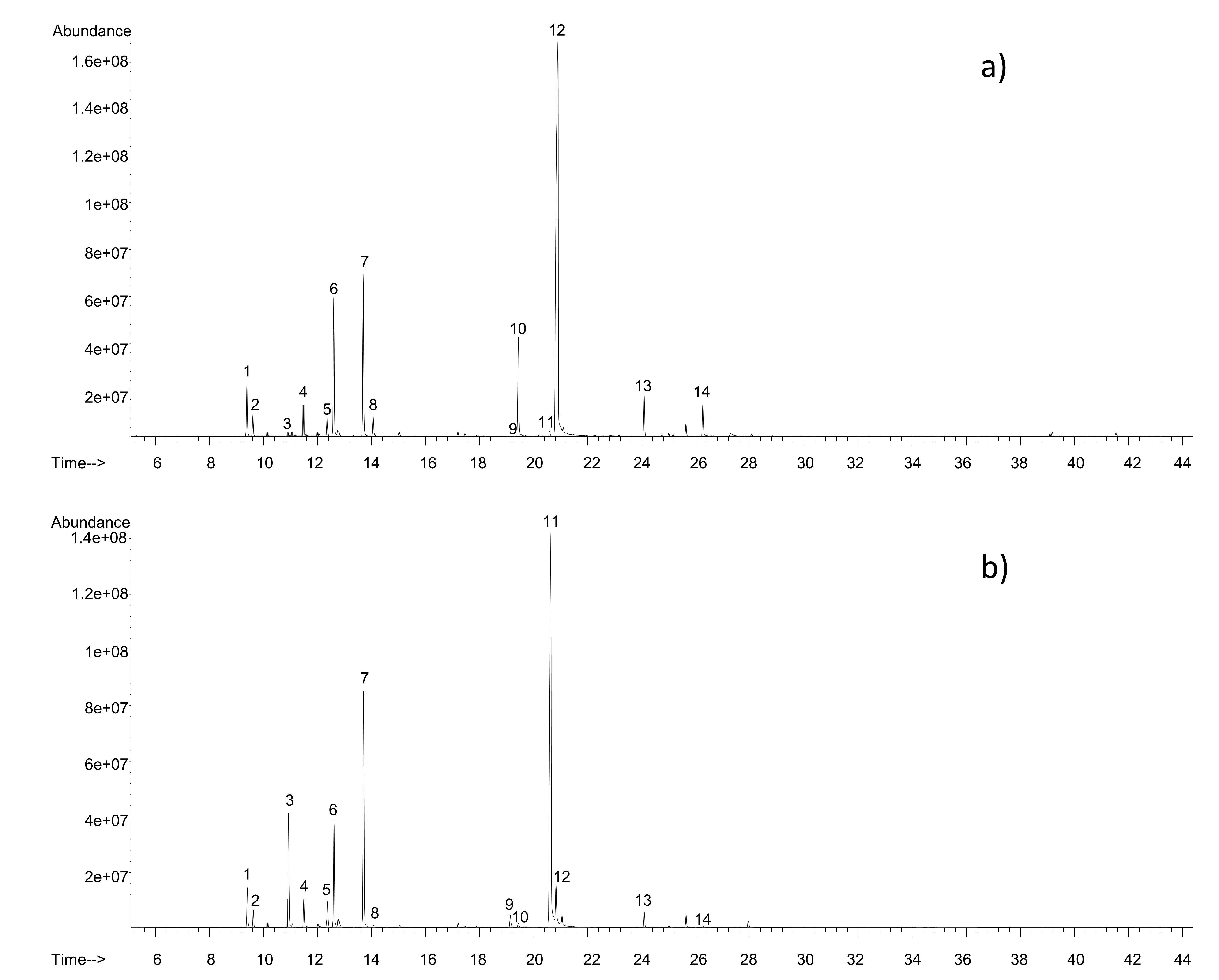

Supplement: Supplementary Figure 4 — Gas chromatography/mass spectrometry (GC/MS) chromatograms of flower volatiles from: (A) a plant representing the carvacrol chemotype and (B) the only plant in the study representing the thymol chemotype. Numbers above peaks indicate the compounds with amount more than 1% in any of the particular samples: (1) α-thujene, (2) α-pinene, (3) sabinene, (4) myrcene, (5) α-terpinene, (6) p-cymene, (7) γ-terpinene, (8) cis-sabinene hydrate, (9) carvacrol methyl ether, (10) thymoquinone, (11) thymol, (12) carvacrol, (13) caryophyllene(E-), and (14) β-bisabolene. [file Figure_4.TIF]

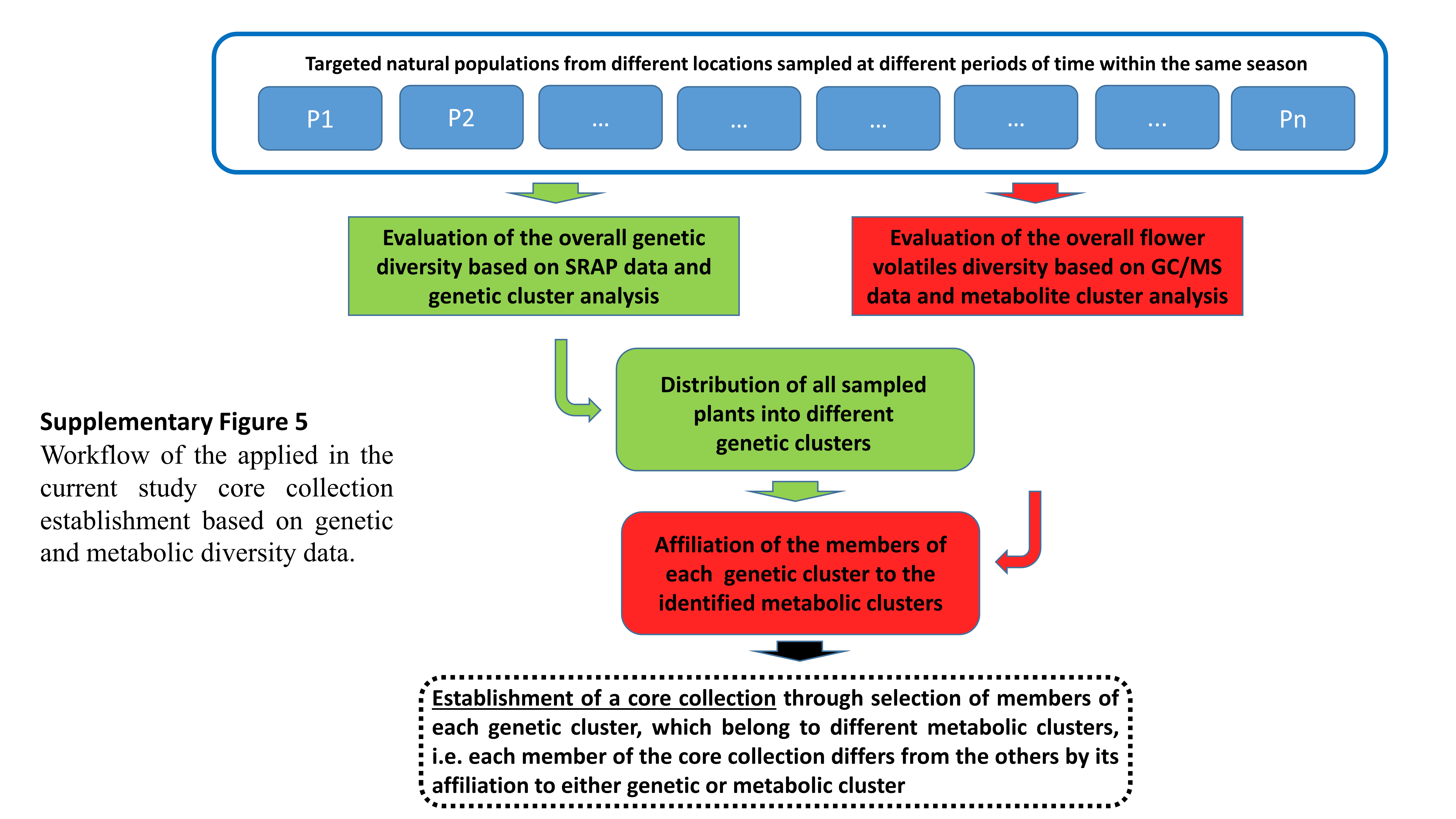

Supplement: Supplementary Figure 5 — Workflow applied for the development of a core collection in the current study based on in parallel evaluation of the genetic and metabolic diversity in natural populations of O. vulgare subsp. hirtum (Link) Ietsw. in Bulgaria. [file Figure_5.TIF]
